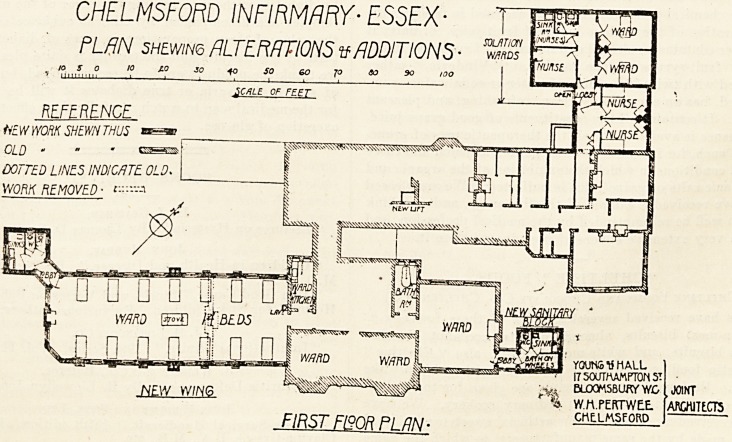# Reconstruction of the Chelmsford Infirmary

**Published:** 1909-08-07

**Authors:** 


### HOSPITAL ADMINISTRATION.

### CONSTRUCTION AND ECONOMICS.

### RECONSTRUCTION OF THE CHELMSFORD INFIRMARY.

The alterations and additions to this hospital, which have recently been completed and were formally opened by Lady Rayleigh on June 8, are the outcome of the advice given to the Governors at the annual meeting in January 1908 by Sir Henry Burdett and Mr. Pearce Gould. Previously to the meeting both gentlemen had been round the hospital with the Chairman, and the result of their inspection was that the existing arrangements were condemned; in particular it was pointed out that the wards were overcrowded to an extent that severely handicapped the patients' chances of recovery; that the sanitary offices were insufficient and not properly disconnected from the wards; that the accommodation for nurses was inadequate and not up to the standard of modern times; that the provision for isolation was ill devised; and that the out-patient department required to be entirely rearranged. The Committee,

GROUND FLOOR PLAN·

FIRST FLOOR PLAN·

convinced of the importance of the points thus raised, determined to give effect to the recommendations which were made on so high an authority, and instructed Mr. Keith Young to draw up a scheme for carrying these recommendations into effect.

The plans show the additions which have been made to the hospital in accordance with the scheme thus prepared.

The hospital, before the alterations were carried out, contained on the ground floor one ward 24 ft. by 21 ft. 6 in., and one 24 ft. by 12 ft., and on the first floor two wards 24 ft. by 21 ft. 6 in. In the rear building was a ward intended for isolation, but generally used for ordinary patients, and a second room which is now the nurses' sitting-room was also used for patients. Two small rooms in the front on the first floor were, and still are used for paying patients.

The only sanitary offices in connection with the wards were w.c.'s leading out of the ward kitchens.

On the south-west side of the front building the end ward was removed and a new ward block two stories high was built.

On the ground floor is a ward for 12 beds, and on the upper floor a ward for 14 beds. The sanitary offices, comprising a large sink-room and two w.c.'s, are placed in a detached block connected with the ward block by cross ventilated bridges. The small room on the ground floor in front, formerly the nurses' sitting-room, is now a ward kitchen, and the back room, formerly the cook's bedroom, is a bath-room. The former ward kitchen, in which was a w.c., has been converted into a linen-room.

The north-east wing could not be enlarged on account of the proximity of the back building; all that was done was to provide a suitable sanitary block properly disconnected. In this block there is a sink-room, a w.c., and space for filling and emptying a wheel bath. The w.c.'s were cleared out of the two ward kitchens in this wing and the ground-floor kitchen converted into a bathroom.

At the back a wing has been built out which contains on the ground floor a new entrance for patients, a large casualty-room, a new dispensary with a medicine waiting-room and patients' exit, and a small dark room attached to the *x*-ray room. The old surgery, with the former entrance thrown in, has been converted into patients' waiting-room, and the old waiting-room is now the consulting-room. The space occupied by a w.c. and lobby and a cupboard has been thrown together to form an examining-room leading out of the consulting-room. The old dispensary, with store-rooms adjoining, now form the *x*-ray room.

On the upper floor the new wing contains two additional bedrooms for nurses and two isolation rooms, with a nurses' room, a w.c., sink-room, and small duty-room. These wards are cut off from the main building by an open lobby.

In this back building the rooms formerly used for patients have now been devoted to the use of nurses, one room overlooking the garden forms a spacious and cheerful sitting-room. The nurses' quarters are now as good as they can be made.

The whole of the work was designed and carried out by Mr. Keith Young (Young and Hall) and Mr. W. H. Pertwee, of Chelmsford, acting in conjunction.

## Figures and Tables

**Figure f1:**
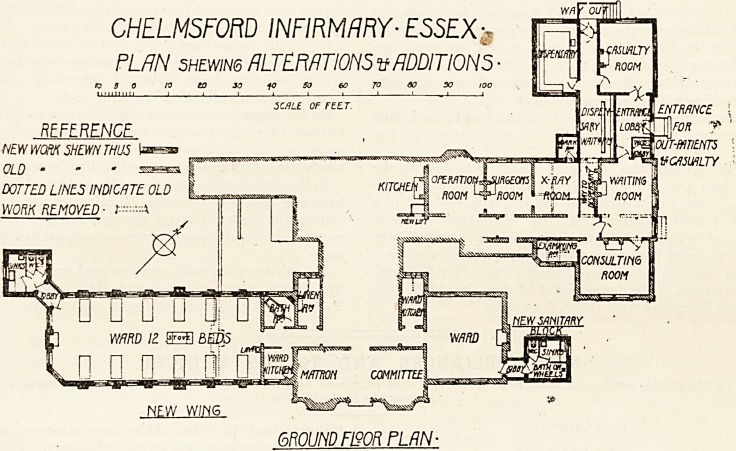


**Figure f2:**